# Respectful maternity care in health centers of Addis Ababa city: a mixed method study

**DOI:** 10.1186/s12884-022-05129-5

**Published:** 2022-10-26

**Authors:** Amaha Haile Abebe, Rose Mmusi-Phetoe

**Affiliations:** Yeroam Consultancy and University of South Africa, Addis Ababa, Ethiopia

**Keywords:** Respectful maternity care, Effective communication, Emotional support, Disrespect and abuse, Privacy and confidentiality

## Abstract

**Objective:**

The study aims to assess quality of obstetric and newborn care including respectfulness of the maternity care.

**Design:**

The study used explanatory sequential mixed methods design .

**Setting:**

This study was conducted in 50 health centres in Addis Ababa city administration January 25 to April 31, 2021.

**Methods:**

During the quantitative phase 500 women in postpartum period were interviewed using structured questionnaire. In the second phase in-depth interview was conducted with 20 midwives and 13 health centre managers. The quantitative data was analysed using Statistical Package for Social Sciences (SPSS). The qualitative data was analysed using Colaizzi’s seven step process.

**Results:**

Only 24.6% of women had respectful maternity care (RMC). Only 46% of women had effective communication during child birth. Only 9.6% of women had emotional support during child birth. Majority of women were encouraged to mobilize, take food and be on labor position of their choice. However, only 22.4 and 18.8% of women respectively had a companion of choice and any pharmacologic pain relief during child birth. One in seven women (15%) had one form of disrespect and abuse during child birth.

**Conclusion:**

Ensuring respectful maternity care needs strong policy direction to health facilities, public education on their right to respectful maternity care, training of care givers and monitoring care through engagement of frontline staff and clients.

## Introduction

World Health Organizations recommended respectful maternity care for a positive childbirth experience. Respectful maternity care is a care organized for and provided to all women in a manner that maintains their dignity, privacy and confidentiality, ensures freedom from harm and mistreatment, and enables informed choice and continuous support during labour and childbirth [1, 2].

Respectful maternity care has been recognized as a core domain of quality obstetric and new-born care which encompasses firstly effective communication - a woman (or her family if required) understands what is happening, what to expect and knows her rights. Secondly, she should receive care with respect and dignity. Thirdly, she should have access to the social and emotional support of her choice [3].

Women’s right to access and receive quality obstetric and newborn care is enshrined in international human right and women’s right charters. The White Ribbon Alliance’s Respectful Maternity Care Charter stated that every woman has the right to information, informed consent and refusal, and respect for her choices and preferences, including companionship during maternity care. Every woman has the right to privacy and confidentiality [4].

However, the rights of women for effective communication, emotional support, dignity and respect during childbirth and post partum period was largely not attained. Many women did not have effective communication with providers and significant proportion of women fail to receive emotional support during childbirth [5].

Every woman has the right to be treated with dignity and respect. Every woman has the right to be free from harm and ill treatment. Every woman has the right to equality, freedom from discrimination, and equitable care. Every woman has the right to liberty, autonomy, self determination, and freedom from coercion [4]. However, 44% of women in sub-Saharan Africa experience at least one form of disrespect and abuse during childbirth in health facilities [6].

Studies in Ethiopia also revealed that significant proportion of women were poorly communicated about the care they received and did not have emotional support for a positive childbirth experience [7, 8]. Half of women face disrespect and abuse during maternity care [9].

This article is part of a large study that examined quality of obstetric care in Health Centers in Addis Ababa city using WHO quality framework [1]. Respectfulness of maternity care was one of the core quality domains assessed. The study aims to develop a strategy to improve quality of obstetric and newborn care including respectfulness of maternity care.

## Research methods

### Study period and setting

The study took place January 25 – December 31, 2021 in 50 health centers of Addis Ababa city the capital of Ethiopia. According to the 2019 Ethiopia mini-demographic and health survey, the city has universal access to health care and respectfully 97 and 96% of pregnant women had Anti-natal care (ANC) and delivery from skilled provider [10].

### Study design and methods

The study used explanatory sequential mixed methods study design. A face to face structured interview with 500 women in post-partum care quantitative phase followed by an in-depth interview with 33 midwives and managers in the qualitative phase. .

### Study population

The study population were all women age 15-49 years old who had delivered babies and were attending immediate post-partum and post-natal care in health centres of Addis Ababa city during the study period and who fulfil the eligibility criteria. Exclusion criteria were:

- Women in the post-partum period who had delivered a baby at home or other health institution because their experience cannot be attributed quality of care at the study health centers
- Women who had childbirth in same health centre but is in the first 6 hours of delivery or is after 6 weeks of delivery, who are very sick or have a sick new-born to take part in this study.

The study population for the qualitative phase which is an in-depth interview were midwives who were heads or deputies of maternity care unit and managers who were age 18 years and above who are working in the health centre at least for 6 months preceding the study. Midwives and managers who work less than 6 weeks and those who were sick or suspected of COVID 19 infection were excluded Midwives who worked for less than 6 weeks do not have enough information about the obstetric and newborn care being provided and those who had or suspected of COVID were excluded because they are sick to interview and reduce risk of transmission to the study team.

### Sample size and sampling technique

The sample size was 500 women in post-partum period who had deliveries in the study health centers and attending immediate postpartum or post-natal care at these health centers. An equal quota of 10 women in the postpartum period was allocated for each of 50 health centres selected for the study to ensure that all the health center quality scores are derived from equal number of women interviewed per health center and are comparable across study health centers. Systematic random sampling was used to select women attending immediate postpartum or post-natal care at the study health centers. The study team had at least 2 days to visit each of the 50 health centres. Based on data from the health centres for the preceding 2 days, the total number of women in the postpartum period eligible for the study expected to visit the health centres in two study days was ranged from 15 to 30 women. The sampling fraction (K) was calculated by dividing the total number of women in the postpartum period expected to be served per health centre in 2 days to the sample of women allocated to each health centre, which is ten women per health centre. The first woman was selected using a lottery method from 1 to K. In facilities with fewer women, we interviewed every woman who fulfilled the eligibility criterion while for some facilities with many postpartum women the sampling fraction (K) was two or three.

The sample size for the in-depth interview were 20 midwives and 13 health center managers and purposeful sampling was applied to select participants for the in-depth interview (Table 1).

**Table 1:** Sampling and sample size for quantitative and qualitative methods

Groups	Sampling method	Sample size	Assumptions
**Quantitative phase sampling and sample size**			
Women in post-partum period	Sampling women survey: 10 women were interviewed from each of the 50 health centres. Systematic random sampling was used to select 10 women who were 6 hours to 6 weeks in the post-partum period. Women were selected from all eligible women who were in immediate post-partum care or who came to the health centre for post-natal care during the 2 -3 days visit to each of the health centres.	**Sample size for women interview** ${n}_1={n}_2=\frac{{\left({Z}_{a/2}\sqrt{2\overline{pq}}+{Z}_{\beta}\sqrt{p_1{q}_1+{p}_2{q}_2}\right)}^2}{\Delta ^2}$ Calculate $\overline{p}=\frac{P_1+{P}_2}{2}$ = 0.25+ 0.15 = 0.2 2 ∆ = p_1_-p_2_ = 0.25 - 0.15 = 0.1 n1 = n2 = (1.96 √.2*.8 + 0.84 √0.25*0.75+ 0.15*0.85)^2^ (0.1)^2^ n1 = 250 n2 = 250 n = n1 + n2 = 500 A study in Addis Ababa reported client satisfaction of 20% on the intra-partum care [11]	P1 = % client satisfied of care at facilities with high quality score = 25% P2 = % client satisfied of care at facilities with low quality score = 15% 95% Confidence interval (Z∝/2 = 1.96) Power 80% (Zβ = 0.84)
**Qualitative phase sampling and sample size**			
Midwife & Manager	Purposeful sampling was applied to select program managers and midwives	20 midwives and 13 health centre managers were purposefully selected	Sample goes until saturation achieved

### Data collection instruments and operational definition

The structured interview questionnaire for Women in the post-partum period was adapted from WHO obstetric and newborn care quality standard [1] and previous studies^7,8.9^ on the topic. The questionnaire was tested for validity using face validity, content validity, criterion related validity and construct validity [12]. The questionnaires were checked at face value if items measure the concept intended to measure. The concept, the conceptual framework, quality statements and quality measures defined in the WHO quality standard for obstetric and newborn care [1] were the basis for development of the study questionnaires, which better ensured that the concept was correctly measured through the survey questionnaires. Panel of experts (four midwives) was used to review list of questions and response option relevance, clarity and completeness to measure the concept of quality and respectfulness of obstetric and newborn care. Reliability is the “consistency” or “repeatability” of measures [12]. In this study Cronbach’s alpha was used to test internal consistency of items in postpartum women’s survey questionnaire where the value was > 0.7 (0.938). The questionnaire was pre-tested in similar setting to test its understandability, and completeness of response options.

A flexible guide was used for the in-depth interview. This study used various measures to ensure trust worthiness of the qualitative phase that includes researcher’s long engagement with the study topic, study setting and participants, triangulation of data sources and methods and members check .

A composite index is a way of compiling one score from a variety of questions or statements that represent an attribute of a phenomenon that cannot be measured with a single question or statement [13]. Respectful maternity care is a concept that cannot be measured with a single question or statement. Therefore, composite index was constructed for effective communication, emotional support and respectful maternity using additive method that sum a number of variables. The following composite indices were developed based on the WHO’s obstetric and newborn care quality framework and standards [1], review of previous studies in Ethiopia [14–16], and a panel of experts. Review of existing literature and the panel of experts (an obstetricians, two midwives and a public health professional) were used to decide on the 75% cut-off point used to define quality and respectful maternity care and weight of items that construct composite index variables.

Effective communication: In this study effective communication embraces greeting and introduction at reception, provision of adequate and timely information in a way woman understand, offering women opportunities to ask questions and an informed consent before an examination. Each of these six indicators was scored ‘1’ when the response is yes and ‘0’ when the response is no. Women were defined to have effective communication when she scored at least 4.5 (score ≥ 75%) out of a maximum of six points.

Emotional support: Six indicators (encouraging women to mobilize, take diet and be in labor position of choice; have companion of choice and who are oriented of their role and pain relief) were used to assess supportiveness of care. Women were defined to have emotional support during childbirth when she scored at least 4.5 (score ≥ 75%) out of a maximum of six points.

Dignified care: Six questions were used to assess whether women had dignified care or not during child birth. Women were defined to have dignified care if she experienced care in an area that is clean and conductive, ensures privacy and confidentiality and did not experience any neglect, disrespect and abuse during childbirth (score 4.5 out of 6).

Respectful Maternity care: eighteen indicators (6 indicators for effective communication, 6 indicators for emotional support and 6 indicators for dignified care) were used to assess whether women had respectful maternity care or not. Women were defined to have respectful maternity care when scored at least 13.5 (≥75%) out of a maximum of eighteen points.

### Data collection

Structured face to face interview with the 500 women in the post-partum period was conducted by four experienced midwives trained for 5 days. Women who fulfil the eligibility criteria were provided information about the study aim, risks, benefits and their rights not to participate or terminate interview any time. Women who agreed to participate signed written consent. Women were interviewed face to face. Each woman was asked of each question on the questionnaire as it reads on the question and recorded responses on the questionnaire.

The researcher conducted all the in-depth interviews at midwives or managers office and privacy was assured. Each participant was provided information using standard information sheet and written informed consent was obtained including for use of the digital voice recorder. The researcher then posed the discussion theme one by one from the discussion guide giving enough time for participants to discuss. The discussion was recoded through digital voice recorder and the researcher have taken note.

### Quantitative data analysis

The data from the questionnaires that have been cleaned were entered to Epi data by a data encoder. Then it was exported from Epi-data to Statistical Package for social sciences (SPSS) version 20 for data coding and analysis. The data was coded, and index variables were constructed. Then the next step was to run descriptive statistics that include frequency (percent or proportion), mean, median mode and standard deviation.

### Qualitative data analysis

In-depth interviews were tape recorded. The researcher, who is a native speaker of the language of interview, transcribed and translated the in-depth interview tape records into English. The qualitative data was analysed using Colaizzi’s seven steps process for phenomenological data analysis. The qualitative data coding was assisted by Atlas ti 5.0, a software for computer aided qualitative data coding. The larger study had four themes and eleven sub themes. One of the theme was respectful maternity care which had three sub-themes - effective communication, emotional support and respectfulness of maternity care.

### Ethical considerations

Ethical clearance was obtained from the ethics committee of the University of South Africa (UNISA) prior to conducting the study. The consent to participate in the study was voluntary. Informed consent was obtained from all subjects and/or their legal guardian(s). All methods were carried out in accordance with relevant guidelines and regulations. All other universal ethical principles relating to research with human subjects were observed.

## Results

### The profiles of the study respondents and participant

The majority (72%) of women were in the age group 20 to 29 years. Mean and median age of women was 26.5 and 26 years respectively (Standard deviation of 4.5 years). The majority, 89%, of women were currently married. The majority (96%) of women had formal mostly elementary or high school education. Most (71%) women were unemployed (Table 2).

**Table 2:** Percentage distribution of women in the postpartum period by Socio-demograpich Characterstics (*N* = 500)

Socio Demograpic Variables	Frequency	Percent
Age		
15-19 year	21	4.2
20-24 year	164	32.8
25-29 year	193	38.6
30-34 year	77	15.4
>/=35 Year	45	9.0
Marital Status		
Never married	47	9.4
Married/living together	447	89.4
Divorced/separated/Widowed	6	1.2
Educational Status		
No Formal education	44	8.8
Primary (grade 1-8)	238	47.6
Secondary (grade 9-12)	138	27.6
College education and above	80	16.0
Employment status		
Employed in Govt, NGO or Private organization	68	13.6
Self Employed	76	15.2
Not Employed	356	71.2
Family Monthly Income based on tax category		
≤ 1650 birr (≤33.00 USD)	73	14.6
1651-3200 birr (33.01-64.00 USD)	192	38.4
3201-5250 birr (64.01-105.00 USD)	117	23.4
≥ 5251 birr (≥105.01 USD)	91	18.2
Not Reported/disclosed	27	5.4
Place of residence		
Within Addis Ababa City	462	92.4
Outside of Addis Ababa city	38	7.6
Number of children alive		
one	218	43.6
Two	167	33.4
Three	77	15.4
Four or More	38	7.6

In-depth interview was conducted with a total of 33 participants whereby twenty were midwives who were maternity unit heads and thirteen were managers. Of the 33 respondents 27 were females and 6 were males. Ten participants under the age of 30 years and 23 participants were 30 years and older. Half of the participants had 5-9 years’ work experience while the rest, that is 12, had 10 years or more work experience. Only 4 participants had work experience less than 5 years.

### Quantitative phase findings

In this study only 24.6% of women had respectful maternity care (RMC).

### Women experience of effective communication during childbirth

The majority (67.6%) of women were greeted by the birth attendant at first encounter during child birth. Only (18%) of women reported that the service providers introduced themselves for them at first encounter during child birth. Respectively, 71 and 72% of women reported being communicated about care received in a way she understands and having enough information about care they received and progress of childbirth. Respectively 69 and 61% of women were given opportunities to ask questions or raise their concerns and were asked of their consent before doing examination or any procedure (Table 3).

**Table 3:** Percentage distribution of women in the post-partum period who experienced effective communication during childbirth (*N* = 500)

Effective communication	Frequency	Percent
The health care provider greeted woman at first encounter during child birth		
No	162	32.4
Yes	338	67.6
The health care staff introduce themselves to the woman at first encounter during child birth		
No	412	82.4
Yes	88	17.6
The health care providers communicated women about the care in a way you understand		
No	146	29.2
Yes	354	70.8
Women was given opportunity to ask questions or raise concerns		
No	154	30.8
Yes	346	69.2
Women receive enough explanation about the care		
No	142	28.4
Yes	358	71.6
Health care providers sought your consent before doing any procedure at all time		
No	193	38.6
Yes	307	61.4
Had effective communication with the obstetric care giver (score ≥ 4.5 out of 6)		
No	270	54.0
Yes	230	46.0

Only 46% of women had effective communication with the obstetric caregivers during child birth (Table 3).

### Women’s experience of emotional support during child birth

The majority (88.6%) of women were encouraged to take enough fluid or fluid diet during child birth. However, only 57.4% of women reported that they were encouraged to mobilize during labour and only 46% of women were encouraged to be in labour position of their choice (Table 4).

**Table 4:** Percentage distribution of women in the post-partum period who experienced emotional support during childbirth (*N* = 500)

Supportive Care	Frequency	Percent
Encouraged to be in labor position of your choice		
No	270	54.0
Yes	230	46.0
Encouraged to mobilize during labor		
No	213	43.6
Yes	287	57.4
Encouraged to have enough fluid or fluid diet during childbirth		
No	57	11.4
Yes	443	88.6
Had her companion of choice during labour and delivery		
No	388	77.6
Yes	112	22.4
Companions were oriented of their role in supporting women during labour and delivery		
No	421	84.2
Yes	79	15.8
Provided pharmacologic pain relief during labour and delivery		
No	406	81.2
Yes	94	18.8
Had emotional support during labor and delivery (score ≥ 4.5 out of 6)		
No	452	90.4
Yes	48	9.6

Only 22.4% of women reported that they had companion of their choice during childbirth and only 18.8% of women received any pharmacologic pain relief during child birth (Table 4).

Only 9.6% of women had emotional support during child birth (Table 4).

### Women’s experience of dignified care during childbirth

The majority (92.2%) of women reported that they had delivered their babies in a clean area. Four-fifth (79.8%) of women reported that labour and delivery care ensure privacy (it is conducted in private room or screen were used during examination). However, only (57%) of women reported that medical information and records were kept confidential (Table 5).

**Table 5:** Percentage distribution of women in the post-partum period who experienced degnified care during childbirth (*N* = 500)

Degnified Care	Frequency	Percent
Childbirth area is clean		
No	39	7.8
Yes	461	92.2
Labor and delivery care you received ensure privacy		
No	101	20.2
Yes	399	79.8
Medical information and records kept confidential		
No	215	43.0
Yes	285	57.0
Women experience at least one form of verbal abuse		
No	439	87.8
Yes	61	12.2
Women experience at least one form of physical abuse		
No	479	95.8
Yes	21	4.2
Women reported that she was being ignored during labor and delivery		
No	477	95.4
Yes	23	4.6
Had dignified care (score ≥ 4.5 out of 6)		
No	146	29.2
Yes	354	70.8

One in seven (15.0%) women had at least one form of disrespect and abuse during childbirth (verbal abuse, physical abuse, being ignored, detained or discriminated). As depicted in Tables 5, 12.2% of women had verbal abuse (an insult, belittling, or threat) during childbirth and 4.2% had physical abuse (hitting, a slap, a pinch, or tied to the bed or harshly forcing leg apart). Similarly, 4.6% of women were reported being ignored by skilled birth attendants during child birth (Table 5).

More than a quarter (29.2%) of women had obstetric and newborn that was not dignified (Table 5).

### Qualitative phase findings

#### Theme −1: effectiveness of communication between clients and providers and the berries

The in-depth interviews also show that it is a routine practice to provide adequate and timely information in a way client understand. However, the way and amount of communication with clients depends on providers skills and the workload. A midwife said.

“*…*. *workload might be a challenge to quality of the communication ….there is also a personal variation some [providers] are skilled communicating women, and some [providers] might lack that skill”*.

#### Theme 2. Emotional support during childbirth and the barriers

In-depth interview revealed that women largely encouraged to mobilize, take fluid diet and to be on labour position of their choice. However, women were not always allowed to have companion of choice during labour and they were not offered pain relief during labour.

A 32 years old midwife said.

*“…We have space problem at the labour ward. We cannot add companions to a space already crowded”*.

Another 27 years old midwife uttered:

*“We do not allow companions due to risk of COVID 19”* (MIDSA)*.*

Generally, women were not provided pharmacologic pain relief during true labour. Lack of national guidance and potent anti-pain drugs (opioid analgesics) were reasons stated for not offering pharmacologic pain relief during child birth.

A 35 years old female midwife said.

*“We know private facilities provide pethidine but in the government facilities we do not have it. We don’t have the guidance to give antipain drugs during labour [true labour]”.*

#### Theme 3. Respectfulness of obstetric and newborn care and the barriers

Most midwives and managers said the obstetric and newborn care ensures respect and dignity of women in labour while a few others feel there were instances where women were facing disrespect and abuse especially during the second stage of labour.

Most said that disrespect and abuse is a thing of the past. These participants noted that training of provides on respectful care, improving awareness of women on their rights and existence of mechanisms to prevent and mitigate disrespect and abuse at health facilities contribute for the improvements. On the other hand, some believe there were instances where providers disrespect and abuse women mostly when women fail to cooperate providers instructions especially during second stage of labour where women get apprehended. A 26 years old Midwife said.

*“We sometimes shout or threaten difficult women who do not cooperate ….”*.

Midwives make effort to ensure privacy of women in labor. However, the fact that there are more than one women per room made ensuring complete privacy a challenge. A 35 years old female medical director said.

*“We take many measures to ensure privacy…. However, the room is narrow and there might be more than one woman. I don’t believe our service ensures complete privacy”*.

## Discussion

Through respectful maternity care is a right of every women only a quarter of women had respectful maternity care. Effective communication is an essential component of experience of quality obstetric and newborn care received by women. Women should receive all information about her care and should feel involved in all decisions taken regarding her treatment [1, 4]. However, Ethiopian women’s right to information and informed consent was the most consistently defiled right during facility-based childbirth. This study further showed that only 46% of women had effective communication with the service providers during childbirth. Similarly, a systematic review in Ethiopia revealed that 16 to 92.5% of women were neither properly communicated nor consented during examination or procedure [17].

This study revealed that high workload, providers communication skills and poor communication culture were factors that influence the quality of client provider communication. This study findings were consistent with a qualitative study on client provider communication in Malawi which reported that workload and providers linguistic and nonverbal communication skills to influence client provider communication [18] .

World Health Organization quality statement underlined that every woman and her family must be provided with emotional support that is sensitive to their needs and strengthens the woman’s capability [1]. However, in this study only a few (9.6%) women had emotional support that strengthen women capability to go through the process of childbirth.

Birth companions provide physical, emotional and spiritual support to women during labor and delivery, thus have a positive impact on the women childbirth experience and birth outcomes [1]. However, only few (22.4%) of women participated in this study were allowed to have companion of their choice during childbirth. This finding is comparable with a study in Arbaminch Southern Ethiopia where only 13.8% of women had companion of their choice during labor and delivery [19]. On the other hand, this study finding was lower than figures reported in other studies [5, 7].

This study identified shortage of space, risk of compromising privacy of laboring women and risk of COVID 19 infection as the major reasons for denying women a companion of their choice during labor. Similarly, a systematic review reported that shortage of space and crowding of labour room in resource limited countries as berries for implementation of the policy of companion of choice during labor [20].

The World Health Organization recommended use of pain relief for women in labor based on her request and preferences [1]. Though there are pharmacologic and non-pharmacologic pain relief options recommended, only few women in developing countries receive pain relief during labor. Though providers in Africa mostly knew about options of pain relief during labor only few were offering pharmacologic pain relief for women in Labor [21, 22]. In this study, only 18.8% of women were receiving any form of pharmacologic pain relief during child birth. Lack of clear guideline and the antipain drugs were reasons for not offering pain pharmacologic relief during child birth. Similar studies done in Nigeria and Ethiopia also reported that lack of skills, shortage of medicines and workload as factors that hinder pain relief during childbirth [21, 22].

The rights of women to be treated with dignity and respect were failed to be realized in developing countries. Systemic review of prevalence studies in Ethiopia revealed that 21.1 to 98.9% of women experience at least one form of disrespect and abuse during childbirth [17]. In this study, more than a quarter of women had a care that is disrespectful or evades privacy and confidentiality and15% of women had at least one form of disrespect and abuse during child birth.

The envisioned limitation of the was the fact that the study used interviews about women experience of child birth and review of patient carts instead of direct observation of clinical care which have minimized objectivity of measurement.

## Conclusion

Ensuring respectful maternity care needs comprehensive interventions that include a policy, guidelines and training curriculum on respectful maternity care. It also needs training of health workers, supportive supervision, mentorship and drill exercises on respectful maternity care skills (effective communication, emotional support and dignified care during child birth). There is a need to conduct public and client education on respectful maternity care through face to face group education at health facilities, electronic media (television and radio) and print media (leaflets, posters, wall charts). Develop clear guidelines on use of pharmacologic and non-pharmacologic pain relief options for women during labor and orient providers. The pharmacologic pain relief options should be always available in adequate quantities at health centers labour and delivery units. All health centers should have a mechanism to receive and investigate and act on client complaints of disrespect and abuse. All health centers should ensure privacy and confidentiality of all women during labor and delivery. All client information and records should always be kept confidential. Health offices and health centers in collaboration with development partners build or renovate additional rooms for maternity care to reduce number of women admitted per room to improve privacy and have space to accommodate companions.

## Data Availability

The datasets used and/or analyzed during the current study are available from the corresponding author on reasonable request.
